# Effect of Free Medicine Distribution on Health Care Costs in Canada Over 3 Years: A Secondary Analysis of the CLEAN Meds Randomized Clinical Trial

**DOI:** 10.1001/jamahealthforum.2023.1127

**Published:** 2023-05-26

**Authors:** Nav Persaud, Michael Bedard, Andrew Boozary, Richard H. Glazier, Tara Gomes, Stephen W. Hwang, Peter Jüni, Michael R. Law, Muhammad Mamdani, Braden Manns, Danielle Martin, Steven G. Morgan, Paul Oh, Andrew D. Pinto, Baiju R. Shah, Frank Sullivan, Norman Umali, Kevin E. Thorpe, Karen Tu, Fangyun Wu, Andreas Laupacis

**Affiliations:** 1 Department of Family and Community Medicine, Temerty Faculty of Medicine, University of Toronto, Toronto, Ontario, Canada; 2 Li Ka Shing Knowledge Institute, St Michael’s Hospital, Unity Health Toronto, Toronto, Ontario, Canada; 3 Department of Family and Community Medicine, St Michael’s Hospital, Unity Health Toronto, Toronto, Ontario, Canada; 4 Institute of Health Policy, Management and Evaluation, University of Toronto, Toronto, Ontario, Canada; 5 Department of Family Medicine, Northern Ontario School of Medicine, Sudbury, Ontario, Canada; 6 Department of Health Policy and Management, Harvard T.H. Chan School of Public Health, Boston, Massachusetts; 7 Dalla Lana School of Public Health, University of Toronto, Toronto, Ontario, Canada; 8 Institute for Clinical Evaluative Sciences, Toronto, Ontario, Canada; 9 Leslie Dan Faculty of Pharmacy, University of Toronto, Toronto, Ontario, Canada; 10 Applied Health Research Centre, St Michael’s Hospital, Unity Health Toronto, Toronto, Ontario, Canada; 11 Department of Medicine, Temerty Faculty of Medicine, University of Toronto, Toronto, Ontario, Canada; 12 Centre for Health Services and Policy Research, School of Population and Public Health, The University of British Columbia, Vancouver, British Columbia, Canada; 13 Centre for Healthcare Analytics Research and Training at St Michael’s Hospital, Toronto, Ontario, Canada; 14 Vector Institute, Toronto, Ontario, Canada; 15 Department of Community Health Sciences, Cumming School of Medicine, University of Calgary, Calgary, Alberta, Canada; 16 Department of Medicine, Cumming School of Medicine, University of Calgary, Calgary, Alberta, Canada; 17 O’Brien Institute for Public Health, Cumming School of Medicine, University of Calgary, Calgary, Alberta, Canada; 18 Libin Cardiovascular Institute, Cumming School of Medicine, University of Calgary, Calgary, Alberta, Canada; 19 Women’s College Hospital Institute for Health Systems Solutions and Virtual Care, Women’s College Hospital, Toronto, Ontario, Canada; 20 School of Population and Public Health, Faculty of Medicine, The University of British Columbia, Vancouver, British Columbia, Canada; 21 Toronto Rehabilitation Institute, University Health Network, Toronto, Ontario, Canada; 22 North York General Hospital, Toronto Western Family Health Team, University Health Network, Toronto, Ontario, Canada; 23 Division of Population and Behavioral Science, School of Medicine, University of St Andrews, St Andrews, Scotland, United Kingdom

## Abstract

**Importance:**

Few interventions are proven to reduce total health care costs, and addressing cost-related nonadherence has the potential to do so.

**Objective:**

To determine the effect of eliminating out-of-pocket medication fees on total health care costs.

**Design, Setting, and Participants:**

This secondary analysis of a multicenter randomized clinical trial using a prespecified outcome took place across 9 primary care sites in Ontario, Canada (6 in Toronto and 3 in rural areas), where health care services are generally publicly funded. Adult patients (≥18 years old) reporting cost-related nonadherence to medicines in the past 12 months were recruited between June 1, 2016, and April 28, 2017, and followed up until April 28, 2020. Data analysis was completed in 2021.

**Interventions:**

Access to a comprehensive list of 128 medicines commonly prescribed in ambulatory care with no out-of-pocket costs for 3 years vs usual medicine access.

**Main Outcome and Measures:**

Total publicly funded health care costs over 3 years, including costs of hospitalizations. Health care costs were determined using administrative data from Ontario’s single-payer health care system, and all costs are reported in Canadian dollars with adjustments for inflation.

**Results:**

A total of 747 participants from 9 primary care sites were included in the analysis (mean [SD] age, 51 [14] years; 421 [56.4%] female). Free medicine distribution was associated with a lower median total health care spending over 3 years of $1641 (95% CI, $454-$2792; *P* = .006). Mean total spending was $4465 (95% CI, −$944 to $9874) lower over the 3-year period.

**Conclusions and Relevance:**

In this secondary analysis of a randomized clinical trial, eliminating out-of-pocket medication expenses for patients with cost-related nonadherence in primary care was associated with lower health care spending over 3 years. These findings suggest that eliminating out-of-pocket medication costs for patients could reduce overall costs of health care.

**Trial Registration:**

ClinicalTrials.gov Identifier: NCT02744963

## Key Points

**Question:**

What is the effect of eliminating out-of-pocket medication costs on total health care costs?

**Findings:**

In this secondary analysis of a randomized clinical trial of 786 primary care patients in Ontario, Canada, eliminating out-of-pocket medication costs was associated with lower reduced total health spending by a median of $1641 and a mean of $4465 over 3 years.

**Meaning:**

These findings suggest that eliminating out-of-pocket medication costs for patients could reduce overall costs of health care.

## Teaser

This secondary analysis of a randomized clinical trial aims to determine the effect of eliminating out-of-pocket medication fees on total health care costs across 9 primary care sites in Ontario, Canada.

## Introduction

Few interventions have been shown to reduce costs in health care.^1^ Health inequities associated with avoidable disparities in outcomes based on income, racism, and other forms of discrimination are estimated to contribute substantially to health spending through poor outcomes for disadvantaged individuals. These inequities can be reduced with better access to basic necessities and health care, including preventive care.^2,3^ The quintuple aim is to reduce costs, improve outcomes, improve patients’ and clinicians’ experiences, and promote health equity.^4^

Cost-related nonadherence to medicines, defined as not taking medicines as instructed because of the cost, is more prevalent among people with low incomes and is associated with poor health outcomes.^5^ The CLEAN Meds (Carefully Selected and Easily Accessible at No Charge Medications) trial examined the effects of free distribution of essential medicines among people experiencing cost-related nonadherence in Ontario, Canada, where physician and hospital care services, but not prescription medications, are universally funded by public payers.^6^ Over 24 months, the CLEAN Meds trial demonstrated that free medicine distribution improved medicine adherence and reduced health care costs.^7^ Free medicine distribution had mixed effects on surrogate health outcomes, reducing systolic blood pressure after 12 months^6^ but not substantially reducing hemoglobin A_1c_ levels, low-density lipoprotein levels, or blood pressure at 24 months.^7^ In this secondary analysis of the CLEAN Meds trial, we report the 36-month results of free medicine distribution on health care costs and health care encounters among primary care patients experiencing cost-related nonadherence.

## Methods

### Design

This was a parallel 2-arm individually randomized clinical trial of patients from 9 primary care sites in Ontario, Canada. After initiation of the trial that was originally planned to last 12 months for each participant, additional funding was obtained to extend the intervention to 36 months, but primary data collection from the control group ended after 24 months.^6^ During the second year of the trial, we decided to compare prespecified outcomes collected passively using health administrative claims data over 36 months. The full study protocol is presented in Supplement 1. The study was reviewed and approved by the Unity Health Toronto Research Ethics Board. Participants provided written informed consent. This report and previous reports follow the Consolidated Standards of Reporting Trials (CONSORT) reporting guideline and include the use of flow diagrams.^8^

### Participants

We included adult (≥18 years old) primary care patients who reported cost-related nonadherence (not filling a prescription or not taking as instructed to make a prescription last longer due to the cost) in the 12 months prior to study participation between June 1, 2016, and April 28, 2017. Primary care clinicians identified potentially eligible patients attending the 6 sites of the St Michael’s Hospital Academic Family Health Team in Toronto, the Assiginack Family Health Team and the Manitoulin Central Family Health Team on Manitoulin Island, and the Huron Shores Family Health Team in Blind River, all in Ontario, Canada. Family members living at the same address of enrolled participants were excluded to reduce contamination. Race and ethnicity were self-reported by participants.

### Trial Procedures

Allocation was concealed using an online application (REDCap [Vanderbilt University]). Randomly permuted blocks of 2 and 4 were used, and randomization was stratified by primary care site. Outcomes were assessed in a blinded fashion, but participants could not be blinded to whether they received free medicine distribution. Usual access to health care services, including outpatient care, laboratory testing, imaging, and inpatient care, was provided to all participants.

Participants allocated to the free medicine distribution group received free access to 128 essential medicines (listed in the eMethods in Supplement 2).^9^ Medicines were mailed to participants in the free distribution group except for time-sensitive medicines such as antimicrobial agents that were dispensed at the point of care. Controlled substances such as opioid analgesics were not included in the intervention, and these medicines, along with other medicines not on the list of essential medicines, could be accessed through usual methods. Participants randomly allocated to the usual access group generally obtained medicines at a community pharmacy. Usual access included out-of-pocket payments for the full cost of medicines, public insurance for social assistance recipients (including those receiving welfare or disability supports), and private insurance.

### Outcomes

Total health care costs were a prespecified secondary outcome of the CLEAN Meds trial, and it is reported as the main focus of this 36-month report. The primary outcome of adherence to appropriately prescribed medicines was previously reported at 12 and 24 months.^6,7^ Health care–related costs were considered from the governmental payer’s perspective. Health care costs were determined using administrative data from Ontario’s single-payer health care system to ascertain the following outcomes: (1) costs of ambulatory visits with primary care physicians, (2) costs of ambulatory visits with specialist physicians, and (3) other physician costs, including laboratory testing, emergency department visits, hospitalizations, publicly funded medications, and home care (eg, home visits by nurses, physiotherapists, and occupational therapists). Publicly funded medicines include medicines covered by public drug plans for social assistance recipients, including those receiving welfare or disability and those older than 65 years. All costs are reported in Canadian dollars with adjustments for inflation. Deaths were also ascertained using administrative data as a potential marker of adverse effects.

We also report health encounter counts. Primary care encounters included visits with family physicians in outpatient settings and walk-in clinic visits. Specialist visits included outpatient visits with consultants such as cardiologists and respirologists.

### Statistical Analysis

We report median (IQR) and mean (SD) health care costs. Differences in means were calculated using a *t* test. Differences in medians were calculated using quantile regression. A χ^2^ test was performed on the number of deaths and the percentage hospitalized. Analyses were conducted at ICES (formerly the Institute for Clinical Evaluative Sciences), with health care administrative data sets that were linked using unique encoded identifiers. SAS Enterprise Guide, version 7.1 (SAS Institute), was used for all analyses. Statistical significance was set at *P* < .05, and we did not correct for multiple comparisons in this secondary analysis.

## Results

**Figure 1.**
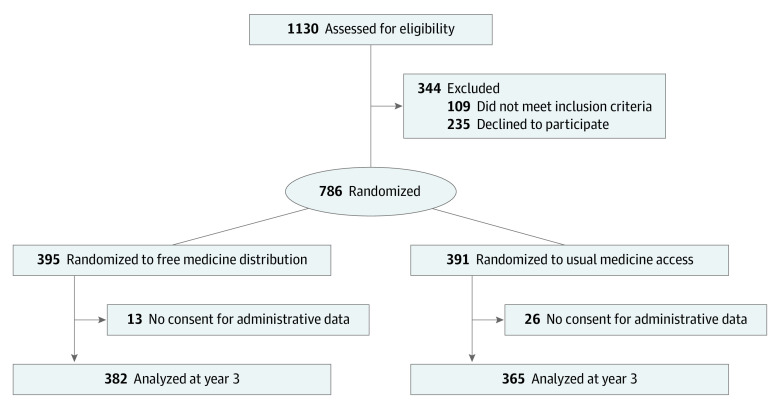
CONSORT Flow Diagram of Participants

A total of 1130 patients were assessed for eligibility, and 786 (mean [SD] age, 51 [14] years; 421 [56.4%] female) were randomly allocated between June 1, 2016, and April 28, 2017. Of those randomized, 39 patients (5.0%) did not consent to the use of health care administrative data, including 13 of 395 patients (3.3%) in the free distribution group and 26 of 391 patients (6.6%) in the usual access group (Figure 1); the characteristics of those included were similar to those randomized (Table 1). This analysis compared 382 patients in the free distribution group and 365 patients in the usual access group. There were 8 deaths (2.1%) in the free medicine distribution group and 14 deaths (3.8%) in the usual access group (*P* = .16).

**Table 1.** Baseline Participant Characteristics by Group

Characteristic	No. (%)			
	Randomized		Included	
	Free distribution (n = 395)	Usual access (n = 391)	Free distribution (n = 382)	Usual access (n = 365)
Sex				
Female	220 (55.7)	219 (56.0)	213 (55.8)	208 (57.0)
Male	175 (44.3)	172 (44.0)	169 (44.2)	157 (43.0)
Age, mean (SD), y	51.0 (14.2)	50.4 (14.3)	50.9 (14.2)	50.2 (14.2)
Age ≥65 y	71 (18.0)	64 (16.4)	65 (17.0)	55 (15.1)
Race and ethnicity				
Black	35 (8.9)	39 (10.0)	32 (8.4)	38 (10.4)
Indigenous	12 (3.0)	14 (3.6)	10 (2.6)	11 (3.0)
Latin American	10 (2.5)	15 (3.8)	10 (2.6)	15 (4.1)
South Asian	25 (6.3)	24 (6.1)	25 (6.5)	22 (6.0)
Southeast or East Asian^a^	28 (7.1)	19 (4.9)	27 (7.1)	18 (4.9)
West Asian^b^	6 (1.5)	5 (1.3)	6 (1.6)	5 (1.4)
White	256 (64.8)	260 (67.5)	249 (65.2)	245 (67.1)
Mixed or other race or ethnicity	22 (5.6)	8 (2.0)	24 (6.3)	7 (1.9)
Declined to provide	1 (0.3)	7 (1.8)	1 (0.3)	5 (1.4)
Main income source				
Wages and salaries (including self-employed)	218 (55.2)	221 (56.5)	215 (56.3)	210 (57.5)
Pension	50 (12.7)	42 (10.7)	51 (13.4)	40 (11.0)
Social support^c^	36 (9.1)	47 (12.0)	33 (8.6)	46 (12.6)
Unemployment insurance	15 (3.8)	9 (2.3)	15 (3.9)	9 (2.5)
Other	56 (14.2)	51 (13.0)	48 (12.6)	45 (12.3)
Declined to provide	20 (5.1)	21 (5.4)	19 (5.0)	10 (2.7)
No. of medicines prescribed at baseline, mean (SD)	5.3 (3.6)	5.6 (4.0)	5.3 (3.6)	5.6 (3.9)
Primary care site				
Urban	269 (68.1)	267 (68.3)	260 (68.1)	247 (67.7)
Rural	126 (31.9)	124 (31.7)	124 (32.5)	120 (32.9)
Prescribed a diabetes treatment	89 (22.5)	91 (23.3)	86 (22.5)	85 (23.3)
Prescribed an antihypertensive	122 (30.9)	114 (29.2)	118 (30.9)	108 (29.6)
Prescribed a statin	81 (20.5)	81 (20.7)	76 (19.9)	75 (20.5)

^a^ Including Chinese, Filipino, Japanese, and Korean.

^b^ Including Arab.

^c^ Welfare or disability.

**Figure 2.**
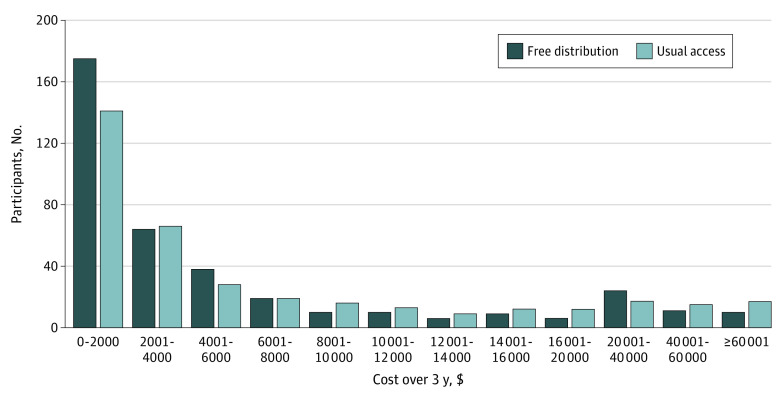
Number of Participants in Each Cost Category Measured Over 3 Years All costs are reported in Canadian dollars with adjustments for inflation.

Eliminating out-of-pocket medication fees was associated with a lower median total health care spending over 3 years of $1641 (95% CI, $454-$2792; *P* = .006; Table 2). Free distribution was associated with a lower number of participants with very high total health care costs (Figure 2). Hospitalizations represented the largest cost in both groups (Table 2). However, between the free distribution and usual access groups, there was not a statistically significant difference in the number of hospitalizations (mean [SD], 0.9 [1.6] vs 1.2 [3.0]; *P* = .08) or the rate of being hospitalized at least once (38.7% vs 44.1%; *P* = .14) (Table 3). There was no statistically significant reduction in primary care visits, specialist visits, and emergency department visits (Table 3). A relatively small number of participants (10 [2.6%] with free distribution and 17 [4.6%] with usual access) had total health care costs greater than $60 000 (Figure 2). Mean total costs were $4465 (95% CI, −$944 to $9874) lower over 3 years, or $1488 (95% CI, −$315 to $3291) lower per year.

**Table 2.** Health Care Spending Over 3 Years by Group of Allocation

Variable	Median (IQR), CAD$		Difference (95% CI), CAD$	Mean (SD), CAD$		Difference (95% CI), CAD$
	Free distribution	Usual access		Free distribution	Usual access	
Primary care visits	58 (0 to 234)	78 (0 to 303)	20 (−16 to 63)	196 (392)	329 (1124)	133 (11 to 255)
Specialist visits	358 (141 to 913)	408 (128 to 1115)	50 (−51 to 152)	730 (1186)	910 (1489)	180 (−14 to 374)
Emergency department visits	275 (0 to 818)	378 (0 to 1249)	103 (−25 to 232)	865 (1776)	1032 (2295)	167 (−129 to 463)
Hospitalizations	0 (0 to 2614)	0 (0 to 4599)	0 (−198 to 198)	6024 (24 322)	8961 (30 266)	2937 (−1019 to 6892)
Publicly funded medicines (excluding cost of intervention)	22 (0 to 1284)	90 (0 to 2426)	68 (−53 to 189)	3441 (12 941)	4225 (12 333)	784 (−1034 to 2601)
Home care	0	0	0^a^	1028 (5303)	982 (6070)	−46 (−866 to 775)
Other (outpatient investigations)	665 (222 to 1436)	645 (286 to 1523)	−20 (−164 to 131)	1097 (1411)	1407 (3509)	310 (−77 to 698)
Total	3098 (1059 to 10 426)	4739 (1506 to 14 719)	1641 (454 to 2792)^b^	13 381 (33 752)	17 846 (41 015)	4465 (−944 to 9874)

^a^ 95% CI not calculated.

^b^ *P* = .006, calculated using quantile regression.

**Table 3.** Health Care Encounters Over 3 Years by Group of Allocation

Variable	Median (IQR)		Difference	Mean (SD)		Difference
	Free distribution	Usual access		Free distribution	Usual access	
Primary care visits	12.0 (6.0 to 20.0)	12.0 (7.0 to 21.0)	0.0 (−1.7 to 1.7)	15.3 (14.7)	17.6 (18.5)	2.3 (−0.1 to 4.7)
Specialist visits	6.0 (2.0 to 11.0)	6.0 (2.0 to 14.0)	0.0 (−1.4 to 1.4)	9.4 (12.4)	11.3 (14.6)	1.9 (0.0 to 3.9)
Emergency department visits	1.0 (0.0 to 3.0)	1.0 (0.0 to 4.0)	0.0 (−0.3 to 0.3)	3.1 (6.1)	3.4 (6.3)	0.3 (−0.6 to 1.2)
Hospitalizations	0.0 (0.0 to 1.0)	0.0 (0.0 to 2.0)	0.0 (−0.2 to 0.2)	0.9 (1.6)	1.2 (3.0)	0.3 (0.0 to 0.6)^a^
≥1 Hospitalization, No. (%)	NA	NA	NA	148 (38.7)	161 (44.1)	5.4 (−1.7 to 12.4)^b^

Abbreviation: NA, not applicable.

^a^ *P* = .08, calculated using *t* test.

^b^ *P* = .14, calculated using χ^2^.

## Discussion

This secondary analysis of a randomized clinical trial found that free medicine distribution to people who have trouble affording medicines was associated with lower total health care spending over 3 years. The largest expenditure was related to hospitalization.

These results build on earlier reports of the CLEAN Meds trial that showed improvements in adherence and health care expenses after 12 and 24 months and mixed effects on surrogate health outcomes.^6,7^ The annual reductions in health care spending were similar at 36 months (mean, $1488; median, $547) relative to the previously reported reductions at 24 months (mean, $1222; median, $558).^7^ This trial involving patients in primary care followed up for 3 years showed a larger benefit than 2 trials of improved medicine access conducted among inpatients followed up for approximately 1 year.^10,11^ These findings suggest that it may take time for adherence to improve health, and preventive medicines might be relatively more beneficial among unselected outpatients than among recently hospitalized patients. The current findings suggest that promoting health equity by improving medicine access can reduce overall health care costs, thereby adding to prior studies showing that free medicine distribution improves the experiences of patients and clinicians.^12,13^ Thus, free medicine distribution may help achieve the quintuple aim that includes both reducing health care spending and improving health equity or fairness in health care and outcomes by ensuring that financial means are not a barrier to realizing the benefits of medicines.^4^ The present findings support a recent call for pharmacoequity, or access to high-quality medicines, “regardless of race and ethnicity, socioeconomic status, or availability of resources”^14^^(p1793)^ by demonstrating economic benefits of fair access. By contrast, eliminating out-of-pocket expenses for all health care services increased overall health care spending in 2 large experimental studies, though other benefits were demonstrated.^15,16^

### Strengths and Limitations

Limitations of the study include an inability to capture expenses related to care accessed outside of Ontario, the lack of consent to the use of health administrative data by some participants, and the lack of information on out-of-pocket expenses. Randomization was stratified by site but, due to an administrative issue related to data sharing, we did not adjust the results for site and, thus, any randomization imbalances within sites that occurred despite stratification could account for observed differences between groups, though there were no statistically significant differences between sites overall. Similarly, we did not adjust the analysis for socioeconomic or clinical factors. This randomized clinical trial of an intervention has clear policy implications, and it provides information about total health care costs using routinely collected administrative data.

## Conclusions

In this secondary analysis of the CLEAN Meds randomized clinical trial, free medicine distribution promoted health equity for patients with cost-related nonadherence and was associated with lower total health care costs.

## Notes

Supplement 1. Trial Protocol

Supplement 2. **eMethods.** List of medicines

Supplement 3. **Nonauthor Collaborators.** The CLEAN Meds study team

Supplement 4. Data Sharing Statement
