# Supplementary material for: Effect of Free Medicine Distribution on Health Care Costs in Canada Over 3 Years: A Secondary Analysis of the CLEAN Meds Randomized Clinical Trial
Source: JAMA Health Forum. 2023 May 26;4(5):e231127. doi: 10.1001/jamahealthforum.2023.1127 (PMC10220517; doi:10.1001/jamahealthforum.2023.1127)
Supplement: Supplement 2. — eMethods. List of medicines [file jamahealthforum-e231127-s002.pdf]

Supplemental Online Content

Persaud N, Bedard M, Boozary A, et al; for the CLEAN Meds study team. Effect of free medicine distribution on health care costs in Canada over 3 years: a secondary analysis of the CLEAN Meds randomized clinical trial. *JAMA Health Forum*. 2023;4(5):e231127. doi:10.1001/jamahealthforum.2023.1127

eMethods. List of medicines

This supplemental material has been provided by the authors to give readers additional information about their work.

© 2023 Persaud N et al. *JAMA Health Forum*.

eMethods. List of medicines

abacavir
acetaminophen
acetylsalicylic acid 325mg
adalimumab
alendronate
allopurinol
amiodarone
amlodipine
amoxicillin
amoxicillin/clavulanic acid
atomoxetine
atorvastatin
azathioprine
azithromycin
baclofen
beclomethasone
benzoyl peroxide
benztropine
bevacizumab
betamethasone
bisoprolol
budesonide
candesartan
carbamazepine
cephalexin
cetirizine
chlorthalidone
ciprofloxacin
clarithromycin
clindamycin
clopidogrel
clotrimazole
cloxacillin
clozapine
conjugated estrogens
dabigatran
dexamethasone
diltiazem
dolutegravir
domperidone
donepezil
doxycycline
efavirenz
eletriptan
emtricitabine
epinephrine
Estradiol p.o and vaginal
ethinylestradiol/levonorgestrel
ferrous fumarate
finasteride
fluconazole
fluoxetine
fluticasone
folic acid
furosemide
fusidic acid
gabapentin
gliclazide
haloperidol
hydrocortisone
hydroxychloroquine
ibuprofen
insulin, long acting
insulin, short acting
ipratropium
labetalol
lamivudine
latanoprost
levodopa/carbidopa
levofloxacin
levonorgestrel - IUD
levothyroxine
lithium
loperamide
medroxyprogesterone
metformin
methimazole
methotrexate
methylprednisolone
metoclopramide
metronidazole
mupirocin
naltrexone
naproxen
nicotine replacement therapy
nitrofurantoin
nitroglycerin
nortriptyline
nystatin
olopatadine
pantoprazole
permethrin
phenytoin
pilocarpine
polyethylene glycol 3350
polymyxin B
potassium
pravastatin
prednisone
propylthiouracil
ramipril
ranitidine
risperidone
rivaroxaban
salbutamol
salicylic acid
salmeterol
senna
sertraline
spironolactone
sulfamethoxazole/ trimethoprim
sulfasalazine
tadalafil
tamsulosin
tenofovir disoproxil fumarate
thiamine
tiotropium
tranexamic acid
tretinoin
trimethoprim
urea
vaginal ring eluting etonogestrel and ethinyl estradiol
valacyclovir
valproic acid
varenicline
vitamin B12
vitamin D
warfarin

© 2023 Persaud N et al. *JAMA Health Forum*.
