# Supplementary material for: Effect of Free Medicine Distribution on Health Care Costs in Canada Over 3 Years: A Secondary Analysis of the CLEAN Meds Randomized Clinical Trial
Source: JAMA Health Forum. 2023 May 26;4(5):e231127. doi: 10.1001/jamahealthforum.2023.1127 (PMC10220517; doi:10.1001/jamahealthforum.2023.1127)
Supplement: Supplement 3. — Nonauthor Collaborators. The CLEAN Meds study team [file jamahealthforum-e231127-s003.pdf]

Supplemental Online Content: Nonauthor Collaborators
*First name, last name, and suffix (if applicable) are required and will appear in PubMed.

*Group Name(s): The CLEAN Meds study team							
***First Name and Middle Initial(s)**	***Last Name**	***Suffix (eg, Jr, III)**	Academic Degrees	Institution	Location (city, state/province, country)	Role or Contribution, eg, chair, principal investigator	Group (if more than 1 Group listed in the byline) and/or Subgroup (eg, Steering Committee)
Nada	Abdel-Malek						
Zoe	von Aesch						
Mouafak	Al Hadi						
Kelly	Anderson						
Gordon	Arbess						
Chris	Barnes						
Peter	Barreca						
Seema	Bhandarkar						
Gary	Bloch						
Tali	Bogler						
Ashna	Bowry						
Donnavan	Boyd						
Marc	Bradford						
Anne	Browne						
Paul	Das						
MaryBeth	Derocher						
Katie	Dorman						
Kathleen	Doukas						
Esther	Ernst						
Allison	Farber						
Hannah	Feiner						
Amy	Freedmon						
Kari	Fulton						
Chantal	Gaudreau						
Abbas	Ghavam-Rassoul						
Rajesh	Girdhari						
Richard	Glazier						
Irv	Gora						
Kimberley	Gordon						

Supplemental Online Content: Nonauthor Collaborators
*First name, last name, and suffix (if applicable) are required and will appear in PubMed.

***First Name and Middle Initial(s)**	***Last Name**	***Suffix (eg, Jr, III)**	Academic Degrees	Institution	Location (city, state/province, country)	Role or Contribution, eg, chair, principal investigator	Group (if more than 1 Group listed in the byline) and/or Subgroup (eg, Steering Committee)
Laurie	Green						
Samantha	Green						
Charlie	Guiang						
Curtis	Handford						
Maryna	Harelnikiva						
Candice	Holmes						
Sue	Hranilovic						
Karl	Igar						
Gwen	Jansz						
Emma	Jeavons						
Nick	Jeeves						
Frances	Kilbertus						
Flo	Kim						
Tara	Kiran						
Holly	Knowles						
Bruce	Kwok						
Sheila	Lakhoo						
Margarita	Lam-Antoniades						
Renata	Leong						
Fok-Han	Leung						
Aisha	Lofters						
Jennifer	McCabe						
Lora	McDougall						
Joanne	Mellan						
Sharon	Mintz						
Matthew	Naccarato						
Maya	Nader						
Kevin	O'Connor						
James	Owen						
Judith	Peranson						
Andrew	Pinto						

Supplemental Online Content: Nonauthor Collaborators
*First name, last name, and suffix (if applicable) are required and will appear in PubMed.

*First Name and Middle Initial(s)	*Last Name	*Suffix (eg, Jr, III)	Academic Degrees	Institution	Location (city, state/province, country)	Role or Contribution, eg, chair, principal investigator	Group (if more than 1 Group listed in the byline) and/or Subgroup (eg, Steering Committee)
Cristina	Pop						
Adam	Pyle						
Julia	Rackal						
Noor	Ramji						
Nasreen	Ramji						
Danyaal	Raza						
Maurianne	Reade						
Jane	Ridley						
Jean	Robinson						
Katherine	Rouleau						
Caroline	Ruderman						
Vanna	Schiralli						
Lee	Schofield						
Mary	Shamas						
Susan	Shepherd						
Rami	Shoucri						
Lenka	Snajdrova						
Andrew	Stadnyk						
Ann	Stewart						
Bill	Sullivan						
Karen	Swirsky						
Joshua	Tepper						
Suzanne	Turner						
Barbara	Vari						
Priya	Vasa						
Karim	Vellani						
Tao	Wang						
William	Watson						
Thea	Weisdorf						
Karen	Weiman						
Sheila	Wijayasinghe						

Supplemental Online Content: Nonauthor Collaborators
*First name, last name, and suffix (if applicable) are required and will appear in PubMed.

***First Name and Middle Initial(s)**	***Last Name**	***Suffix (eg, Jr, III)**	Academic Degrees	Institution	Location (city, state/province, country)	Role or Contribution, eg, chair, principal investigator	Group (if more than 1 Group listed in the byline) and/or Subgroup (eg, Steering Committee)
Jean	Wilson						
Patricia	Windrim						
