# Supplementary material for: Effect of Free Medicine Distribution on Health Care Costs in Canada Over 3 Years: A Secondary Analysis of the CLEAN Meds Randomized Clinical Trial
Source: JAMA Health Forum. 2023 May 26;4(5):e231127. doi: 10.1001/jamahealthforum.2023.1127 (PMC10220517; doi:10.1001/jamahealthforum.2023.1127)
Supplement: Supplement 4. — Data Sharing Statement [file jamahealthforum-e231127-s004.pdf]

Data Sharing Statement

Persaud. Effect of Free Medicine Distribution on Health Care Costs in Canada Over 3 Years. *JAMA Health Forum*. Published May 26, 2023. doi:10.1001/jamahealthforum.2023.1127

Data

Data available: We have publicly posted anonymized individual patient level data: https://figshare.com/articles/dataset/CLEAN_Meds_trial_Year_2_data/14428832
